# Nintedanib combined with immunosuppressive agents improves forced vital capacity in connective tissue disease-associated PF-ILD: a single-center study

**DOI:** 10.1186/s41927-024-00400-y

**Published:** 2024-06-18

**Authors:** Yusuke Ushio, Risa Wakiya, Tomohiro Kameda, Shusaku Nakashima, Hiromi Shimada, Taichi Miyagi, Koichi Sugihara, Rina Mino, Mao Mizusaki, Kanako Chujo, Ryoko Kagawa, Hayamasa Yamaguchi, Norimitsu Kadowaki, Hiroaki Dobashi

**Affiliations:** Faculty of Medicine, Division of Hematology, Rheumatology and Respiratory Medicine, Department of Internal Medicine, Kagawa University, 1750-1 Ikenobe, Miki-cho, Kita- gun, Kagawa, Kagawa 761-0793 Japan

**Keywords:** Connective tissue disease (CTD), Interstitial lung disease (ILD), Progressive fibrosing interstitial lung disease (PF-ILD), Nintedanib, Antifibrotic therapy

## Abstract

**Background:**

In 2020, Nintedanib (NTB), a tyrosine kinase inhibitor, was the first drug approved worldwide for treating progressive fibrosing interstitial lung disease (PF-ILD). This study evaluated the efficacy and safety of NTB in Japanese patients with CTD-associated PF-ILD in a real-world setting, as there are few reports on this topic. We also evaluated the efficacy and safety of combination therapy with NTB and immunosuppressive agents (IS).

**Methods:**

CTD-associated PF-ILD patients receiving NTB at our institution were included in this retrospective study. To evaluate the efficacy and safety of NTB, we investigated changes in forced vital capacity (FVC) (%), diffusing capacity for carbon monoxide (DLCO) (%), monthly change in FVC (%/month), serum Krebs von den Lungen-6 (KL-6) levels (U/mL) before and after NTB treatment, and adverse events (AEs) during NTB treatment. Moreover, to evaluate the efficacy of the NTB + IS combination therapy, we divided the patients into two groups: one received only NTB (NTB group), and the other received both NTB and IS (NTB + IS group) following the diagnosis of CTD-associated PF-ILD. We analyzed the differences in the changes of these variables between the two groups.

**Results:**

Twenty-six patients with CTD-associated PF-ILD were included. After NTB treatment, there were no significant deteriorations in FVC (%) and DLCO (%), while the monthly change in FVC (%/month) significantly increased (*p* < 0.001). The changes in FVC (%) and the monthly change in FVC (%/month) were significantly greater in the NTB + IS group than in the NTB group. Following NTB treatment, the mean serum KL-6 levels significantly decreased (*p* < 0.001). AEs associated with NTB in this study were similar to those in previous clinical trials, and there was no significant difference in the incidence of AEs between the two groups.

**Conclusions:**

This study demonstrates that NTB is an effective medication for slowing the progression of CTD-associated PF-ILD in real-world settings. NTB + IS combination therapy for CTD-associated PF-ILD may be more effective than NTB alone in slowing the progression of CTD-associated PF-ILD.

**Supplementary Information:**

The online version contains supplementary material available at 10.1186/s41927-024-00400-y.

## Background

In recent years, a subgroup of ILDs characterized by a progressive decline in pulmonary function, increased fibrosis on high-resolution chest computed tomography (HRCT), and worsening respiratory symptoms has been identified as progressive fibrosing ILD (PF-ILD), regardless of the underlying disease [1]. The clinical course of PF-ILD, which progresses despite appropriate treatment, is known to have a poor prognosis, similar to that of untreated idiopathic pulmonary fibrosis (IPF) [2].

PF-ILD is also found in connective tissue disease-associated ILD (CTD-ILD); it progresses in 40% of cases of rheumatoid arthritis-associated ILD (RA-ILD), 32% of systemic sclerosis-associated ILD (SSc-ILD), 16% of polymyositis/dermatomyositis-associated ILD (PM/DM-ILD), and 24% of Sjögren’s syndrome-associated ILD (SS-ILD) [3]. Treatment for CTD-associated PF-ILD is an important issue that needs to be addressed.

In 2020, Nintedanib (NTB), a tyrosine kinase inhibitor, was the first drug approved worldwide for the treatment of PF-ILD [1, 4]. In a randomized, double-blind, placebo-controlled, Phase III trial (INBUILD trial), the annual reduction in forced vital capacity (FVC) in the NTB group was significantly lower than that in the placebo group. This supports the efficacy of NTB in slowing the progression of PF-ILD [1] and suggests similar efficacy in CTD-associated PF-ILD in this subgroup [5].

However, there are few reports on the efficacy and safety of NTB for treating PF-ILD associated with CTD in real-world settings. Furthermore, although the concomitant use of immunosuppressive agents (IS) was limited within 6 months of starting NTB in the INBUILD trial [1], many IS are recommended as the standard treatment for CTD-ILD [6–10]. When CTD-associated PF-ILD is diagnosed, it is unclear whether using NTB alone or combining NTB with IS is more effective in slowing the progression of CTD-associated PF-ILD.

The study evaluated the efficacy and safety of NTB in Japanese patients with CTD-associated PF-ILD in a real-world setting. We also investigated whether there were differences in efficacy and safety between the NTB group and the NTB + IS combination group.

## Methods

### Patients

Patients with CTD-associated PF-ILD who received NTB at Kagawa University Hospital in Japan from May 1, 2020, to September 30, 2023, were included in this retrospective study. Patients who did not undergo pulmonary function tests (PFTs) at the start of NTB treatment (within 3 months before starting NTB) and after NTB treatment (6–12 months after starting NTB) were excluded.

The diagnosis of CTD was established based on the following criteria: RA, the American College of Rheumatology (ACR)/European League Against Rheumatism (EULAR) classification criteria for RA (2010) [11]; SS, the revised criteria by the Japanese Ministry of Health for SS (1999) [12]; SSc, the ACR/EULAR classification criteria for SSc (2013) [13]; PM/DM, the ACR/EULAR classification criteria for idiopathic inflammatory myopathies (2017) [14]; ANCA-associated vasculitis (microscopic polyangiitis (MPA), granulomatosis with polyangiitis, eosinophilic granulomatosis with polyangiitis (EGPA)), the European Medicines Agency algorithm (2007) [15].

Patients had to meet at least one of the following criteria for PF-ILD [1] to be eligible for starting receiving NTB: a relative decline in FVC of at least 10% of the predicted value within the 24 months prior, a relative decline in FVC of 5% to less than 10% of the predicted value accompanied by worsening respiratory symptoms or an increased extent of fibrosis on HRCT, or worsening respiratory symptoms and an increased extent of fibrosis on HRCT.

### Data collection

We collected the following information from the patients’ medical records: demographic information (gender, age), smoking history, imaging patterns of ILD on HRCT, primary CTD, duration of CTD-ILD, concomitant IS, PFT data (vital capacity (VC) (percent of predicted value, %), FVC (percent of predicted value, %), forced expiratory volume in 1 s divided by FVC (FEV1/FVC) (%), diffusing capacity for carbon monoxide (DLCO) (percent of predicted value, %), DLCO divided by alveolar volume (DLCO/VA) (percent of predicted value, %)) before NTB treatment (within 24 months before the start of NTB), at the beginning of NTB treatment (within 3 months before the start of NTB), and after NTB treatment (6–12 months following the start of NTB), serum Krebs von den Lungen-6 (KL-6) (U/mL), a biomarker indicating the activity level of CTD-ILD [16], measured closest to the time PFT was conducted, and adverse events (AEs) during NTB treatment.

To assess the efficacy and safety of NTB, we investigated changes in FVC (%), DLCO (%), monthly changes in FVC (%/month), and serum KL-6 (U/mL) before and after NTB treatment, the association between changes in serum KL-6 (U/mL) and changes in FVC (%), and AEs during NTB treatment.

To clarify the efficacy and safety of the combination therapy with NTB and IS for CTD-associated PF-ILD, we divided the patients into two groups: one received only NTB (NTB group) and the other received both NTB and IS (NTB + IS group) following the diagnosis of CTD-associated PF-ILD. In the NTB + IS group, additional IS is defined as glucocorticoids (GC) at a dosage of ≥ 20 mg/day of prednisolone or equivalent, and newly initiated IS following the diagnosis of CTD-associated PF-ILD. We analyzed the differences in changes in FVC (%), DLCO (%), monthly changes in FVC (%/month), and KL-6 (U/mL), as well as the incidence of AEs between the NTB group and the NTB + IS group.

### Statistical analysis

Differences in FVC (%), DLCO (%), monthly change in FVC (%/month), and KL-6 (U/mL) before and after NTB treatment were analyzed using the Wilcoxon signed rank test. The relationship between changes in serum KL-6 levels (U/mL) and changes in FVC levels (%) was analyzed using Spearman’s correlation coefficient.

To compare the NTB group with the NTB + IS group, we analyzed the differences in changes in FVC (%), DLCO (%), monthly change in FVC (%/month), and KL-6 (U/mL) between the two groups using Wilcoxon’s rank sum test. Additionally, we examined the differences in the incidence of AEs between the two groups using Fisher’s exact test.

All p-values were two-sided, and a p-value < 0.05 was considered significant. The data were analyzed using JMP® Pro 14 software (SAS Institute, Cary, USA).

## Results

### Patient characteristics

Twenty-six patients with CTD-associated PF-ILD were included in this study. Patient characteristics are shown in Table 1. The mean age was 65.1 years, with 16 participants (61.5%) being female, and 11 (42.3%) having a history of smoking. The primary CTDs were SSc in 11 patients (42.3%), PM/DM in 6 (23.1%), RA in 4 (15.4%), MPA in 3 (11.5%), EGPA in 1 (3.8%), and SS in 1 (3.8%). The mean duration of CTD-ILD was 8.2 years.

**Table 1:** Patient characteristics

Characteristics	Total (*n* = 26)	NTB group (*n* = 15)	NTB + IS group (*n* = 11)	*p*
Age, years	65.1 ± 9.8	62.5 ± 9.5	68.5 ± 9.6	0.113
Sex, Female	16 (61.5)	9 (60.0)	7 (63.6)	1.000
Former or current smoker	11 (42.3)	6 (40.0)	5 (45.5)	1.000
UIP-like fibrotic pattern on high-resolution CT	14 (53.9)	9 (60.0)	5 (45.5)	0.692
Primary CTD				
Systemic sclerosis (SSc)	11 (42.3)	8 (53.3)	3 (27.3)	0.246
Polymyositis, Dermatomyositis (PM/DM)	6 (23.1)	3 (20.0)	3 (27.3)	1.000
Rheumatoid arthritis (RA)	4 (15.4)	2 (13.3)	2 (18.2)	1.000
Microscopic polyangiitis (MPA)	3 (11.5)	0 (0)	3 (27.3)	0.064
Eosinophilic granulomatosis with polyangiitis (EGPA)	1 (3.8)	1 (6.7)	0 (0)	1.000
Sjögren’s syndrome (SS)	1 (3.8)	1 (6.7)	0 (0)	1.000
Duration of CTD, years	9.8 ± 8.3	9. 0 ± 6.4	6.1 ± 5.2	0.232
Duration of CTD-ILD, years	7.7 ± 5.6	9. 0 ± 6.4	5.9 ± 3.9	0.299
Criteria for disease progression over the past 24 months				
Relative decline in FVC of ≥ 10% of the predicted value	7 (26.9)	3 (20.0)	4 (36.4)	0.407
Relative decline in FVC of ≥ 5% to ≤ 10% of the predicted value, along with worsening respiratory symptoms or increased extent of fibrosis on high-resolution CT.	6 (23.1)	4 (26.7)	2 (18.2)	1.000
Worsening respiratory symptoms and increased extent of fibrosis on high-resolution CT	13 (50.0)	8 (53.3)	5 (45.5)	1.000
Additional immunosuppressive agents administered following the diagnosis of CTD-associated PF-ILD				
Rituximab	6 (24.0)	0 (0)	6 (54.6)	0.002^**^
Glucocorticoids (≥ 20 mg/day of prednisolone equivalent)	4 (15.4)	0 (0)	4 (36.4)	0.022*
Abatacept	2 (7.7)	0 (0)	2 (18.2)	0.169
Cyclophosphamide	1 (3.8)	0 (0)	1 (9.1)	0.423
Mycophenolate mofetil	1 (4.0)	0 (0)	1 (9.1)	0.423
Tacrolimus	1 (4.0)	0 (0)	1 (9.1)	0.423

Data are presented as means ± standard deviation (SD) or as numbers (%), unless otherwise indicated. UIP: usual interstitial pneumonia; CT: computed tomography; CTD: connective tissue diseases; ILD: interstitial lung disease

For statistical analyses, *p < 0.05, **p < 0.01. P-value: Wilcoxon signed rank test, Fisher’s exact test

After being diagnosed with CTD-associated PF-ILD, 15 out of the 26 patients received only NTB (NTB group), while the remaining 11 patients received both NTB and IS (NTB + IS group). In the NTB + IS group of 11 patients, the most frequently used additional IS was rituximab (RTX), administered to 6 patients (54.6%), followed by glucocorticoids (GC), administered to 4 patients (36.4%).

### Impact of NTB treatment on pulmonary function

The mean FVC (%) and mean DLCO (%) for all patients at the start of NTB treatment were 62.5% and 48.1%, respectively. In the 18 patients who had undergone PFT within 24 months prior to starting NTB treatment, the mean monthly change in FVC (%/month) before starting NTB was 0.70%/month (Table 2).

**Table 2:** Pulmonary function and laboratory data before and after NTB treatment

Pulmonary function data / Laboratory data	Total (*n* = 26)	NTB group (*n* = 15)	NTB + IS group (*n* = 11)	*p*
At the start of NTB treatment				
VC (vital capacity)				
% of predicted value, %	64.8 ± 17.0	65.8 ± 18.8	63.4 ± 14.9	0.876
mean value, mL	1,860.4 ± 709.2	1,965.3 ± 816.3	1,717.3 ± 534.5	0.378
FVC (forced vital capacity)				
% of predicted value, %	62.5 ± 16.7	64.3 ± 18.7	60.1 ± 14.0	0.586
FEV1/FVC (forced expiratory volume in 1 s divided by forced vital capacity)				
mean value, %	89.4 ± 6.8	86.6 ± 6.7	93.2 ± 5.1	0.020^*^
DLCO (diffusing capacity for carbon monoxide) †				
% of predicted value, %	48.1 ± 24.3 (*n* = 15)	56.4 ± 24.4 (*n* = 8)	38.7 ± 22.1 (*n* = 7)	0.272
DLCO/VA (diffusing capacity for carbon monoxide divided by alveolar volume) †				
% of predicted value, %	71.9 ± 27.0 (*n* = 15)	76.6 ± 20.2 (*n* = 8)	66.6 ± 34.2 (*n* = 7)	0.524
Monthly change in FVC before NTB treatment§, %/month	−0.70 ± 0.56 (*n* = 18)	−0.46 ± 0.35 (*n* = 11)	−1.07 ± 0.65 (*n* = 7)	0.024^*^
KL-6, U/mL	1,240.1 ± 839.7	1,001.7 ± 661.5	1,565.2 ± 974.1	0.040^*^
After NTB treatment				
Follow-up period since NTB was started, months	8.4 ± 2.3	8.1 ± 2.4	8.8 ± 2.0	0.351
VC (vital capacity)				
% of predicted value, %	67.9 ± 18.5	64.0 ± 19.1	73.3 ± 17.1	0.276
mean value, mL	1,941.9 ± 760.4	1,908.0 ± 823.3	1,988.2 ± 701.7	0.917
FVC (forced vital capacity)				
% of predicted value, %	67.0 ± 17.2	63.2 ± 17.6	72.3 ± 15.8	0.213
FEV1/FVC (forced expiratory volume in 1 s divided by forced vital capacity)				
mean value, %	86.5 ± 9.5	84.5 ± 10.9	89.3 ± 6.6	0.213
DLCO (diffusing capacity for carbon monoxide) †				
% of predicted value, %	46.0 ± 24.0 (*n* = 15)	50.6 ± 28.0 (*n* = 8)	40.7 ± 19.3 (*n* = 7)	0.643
DLCO/VA (diffusing capacity for carbon monoxide divided by alveolar volume) †				
% of predicted value, %	68.4 ± 24.4 (*n* = 15)	69.3 ± 26.0 (*n* = 8)	67.3 ± 24.5 (*n* = 7)	0.772
Monthly change in FVC after NTB treatment^§^, %/month	+ 0.54 ± 1.56 (*n* = 18)	−0.17 ± 0.86 (*n* = 11)	+ 1.50 ± 1.81 (*n* = 7)	0.001**
KL-6, U/mL	964.4 ± 597.2	883.3 ± 518.7	1,075.1 ± 974.1	0.468

Data are expressed as mean ± standard deviation (SD). NTB: nintedanib; IS: immunosuppressive agents; KL-6: Krebs von den Lungen-6

† DLCO was measured in 15 of 26 patients

§ The monthly change in FVC (%/month) before and after NTB treatment was calculated for 18 of the 25 patients who underwent a pulmonary function test before NTB treatment (within 24 months before the start of NTB), at the start of NTB treatment (within 3 months before the start of NTB), and after NTB treatment (6–12 months after the start of NTB).

For statistical analyses, *p < 0.05, **p < 0.01. P-value: Wilcoxon signed rank test, Fisher’s exact test

There was no difference in FVC (%) and DLCO (%) at the start of NTB treatment between the NTB and NTB + IS groups. However, the monthly decrease in FVC (%/month) before starting NTB treatment was significantly greater in the NTB + IS group than in the NTB group (− 1.07% ± 0.65%/month vs. −0.46% ± 0.35%/month, *p* = 0.024) (Table 2).

**Fig. 1:**
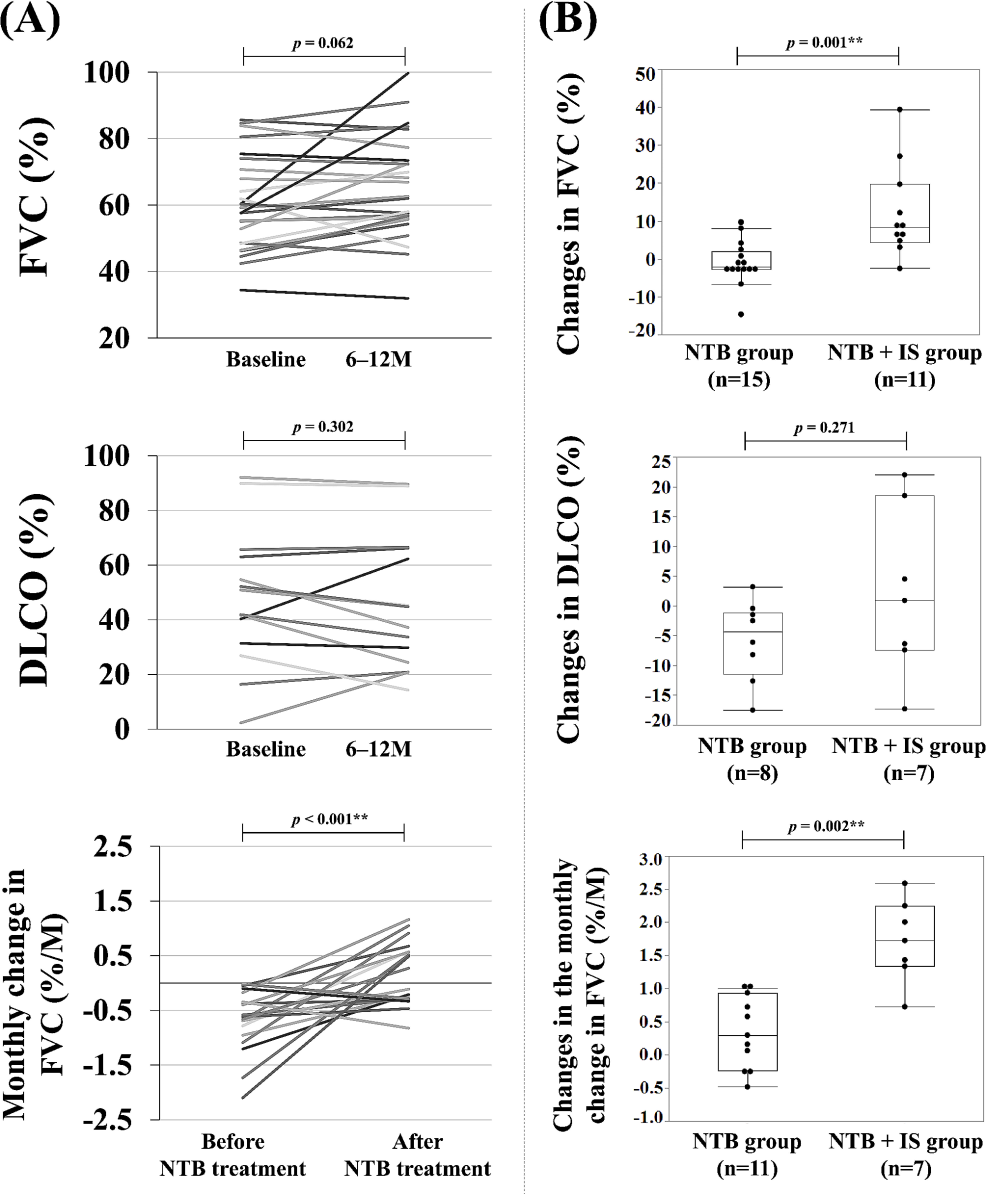
(**A**) Pulmonary function before and after NTB treatment. (**B**) Differences in pulmonary function changes between the NTB group and the NTB + IS group. FVC, forced vital capacity; DLCO, diffusing capacity for carbon monoxide; NTB, nintedanib; IS, immunosuppressive agents. For statistical analyses, *p < 0.05, **p < 0.01. P-value: Wilcoxon signed rank test, Wilcoxon rank sum test

After NTB treatment, PFT follow-up occurred at a mean of 8.4 months. The mean FVC (%) and DLCO (%) in all patients following NTB treatment were 67.0% and 46.0%, respectively (Table 2). Although the increase in FVC (%) was not statistically significant, there was a significant monthly change in FVC (%/month), with a change from − 0.70% ± 0.56%/month to + 0.54% ± 1.56%/month (Fig. 1A). In the comparison between the NTB group and the NTB + IS group, changes in FVC (%) and the monthly change in FVC (%/month) were all significantly greater in the NTB + IS group than in the NTB group (+ 12.1% vs. −1.1%, + 1.71%/month vs. +0.34%/month, respectively) (Fig. 1B).

### Effect of NTB treatment on serum KL-6 levels

The mean serum KL-6 level in all patients at the start of NTB treatment was 1,240.1 U/mL, being significantly higher in the NTB + IS group than in the NTB group (1,565.2 U/mL vs. 1,001.7 U/mL, *p* = 0.040) (Table 2).

**Fig. 2:**
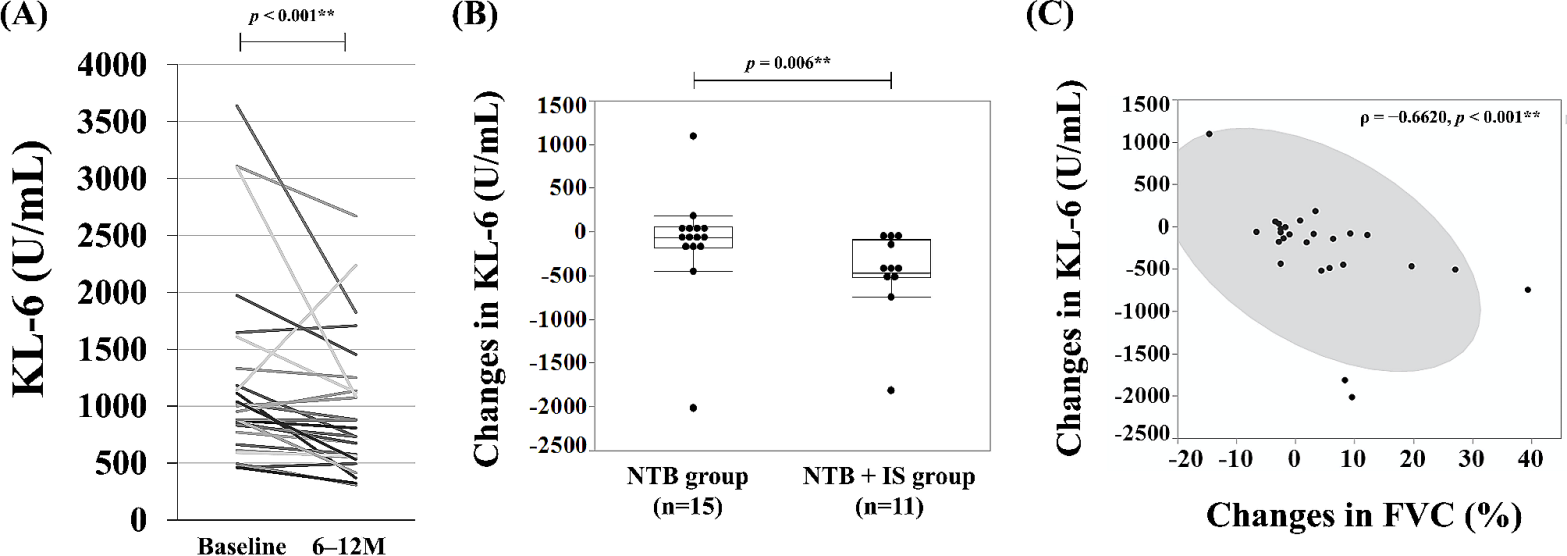
(**A**) Serum KL-6 levels before and after NTB treatment. (**B**) Differences in serum KL-6 level changes between the NTB group and the NTB + IS group. (**C**) Association between changes in serum KL-6 levels (U/mL) and changes in FVC (%) is being examined. KL-6, Krebs von den Lungen-6; FVC, Forced Vital Capacity; NTB, Nintedanib; IS, Immunosuppressive Agents. For statistical analyses, *p < 0.05, **p < 0.01. *P*-value: Wilcoxon signed rank test, Wilcoxon rank sum test

After NTB treatment, the mean serum KL-6 levels in all patients decreased significantly (before, 1,240.1 U/mL; after, 964.4 U/mL; *p* < 0.001). Furthermore, changes in serum KL-6 levels were significantly greater in the NTB + IS group than in the NTB group (− 490.1 U/mL vs. −118.5 U/mL, *p* = 0.006) (Fig. 2A, B). A significant negative correlation was observed between changes in serum KL-6 (U/mL) and changes in FVC (%) (ρ = −0.6620, *p* < 0.001) (Fig. 2C).

### AEs during NTB treatment

Fourteen patients (53.9%) had diarrhea, two (7.7%) elevated liver enzymes, one (3.9%) nausea, and one (3.9%) headache. The dose of NTB was reduced in 11 patients (42.3%), with diarrhea being the most common reason for the dose reduction. No patients discontinued NTB due to AEs. In the NTB + IS group, one patient (9.1%) had gastrointestinal perforation, a serious AE (Table 3).

**Table 3:** AEs during NTB treatment

Adverse events (AEs)	Total (*n* = 26)	NTB group (*n* = 15)	NTB + IS group (*n* = 11)	*P*
Diarrhea	14 (53.9)	9 (60.0)	5 (45.5)	0.692
Elevated liver enzymes	2 (7.7)	2 (13.3)	0 (0)	0.492
Nausea	1 (3.9)	0 (0)	1 (9.1)	0.423
Headache	1 (3.9)	0 (0)	1 (9.1)	0.423
Gastrointestinal perforation	1 (3.9)	0	1 (9.1)	0.423
Dose reduction	11 (42.3)	6 (40.0)	5 (45.5)	1.000
Reasons for dose reduction				
Diarrhea	8 (30.8)	4 (26.7)	4 (36.4)	0.683
Elevated liver enzymes	2 (7.7)	2 (13.3)	0 (0)	0.492
Nausea	1 (3.9)	0	1 (9.1)	0.423
Discontinuation	0 (0)	0 (0)	0 (0)	―

Data are expressed as number (%). NTB: nintedanib; IS: immunosuppressive agents

For statistical analyses, *p < 0.05, **p < 0.01. *P*-value: Fisher’s exact test

## Discussion

CTD-associated PF-ILD is a significant complication in terms of mortality and morbidity. However, there are few reports on the use of NTB treatment for CTD-associated PF-ILD in real-world settings. The sole publication is a case series from Greece involving 21 patients with CTD-associated PF-ILD, of whom 13 underwent PFT before and after NTB treatment. This study reported that NTB is effective in slowing the progression of ILD [17]. In this study, we investigated patients with CTD-associated PF-ILD who received NTB at our institution. After starting NTB treatment, FVC (%) and DLCO (%) remained stable, while the monthly change in FVC (%/M) significantly increased. This indicates that NTB is an effective medication for slowing the progression of CTD-associated PF-ILD in a real-world setting.

Additionally, when comparing the NTB group with the NTB + IS group, the FVC increased significantly more in the NTB + IS group than in the NTB group. These results suggest that combining NTB with IS may be more effective than NTB alone in slowing the progression of CTD-associated PF-ILD.

A subanalysis of the SENSCIS trial, which assessed the efficacy of NTB in treating SSc-ILD, demonstrated the efficacy of NTB in combination with mycophenolate mofetil [18]. However, evidence is lacking for the combined use of NTB with IS other than MMF.

The predominant CTD in our population was SSc, similar to the distribution of participants in the INBUILD trial. However, all three patients with SSc-ILD in the NTB + IS group received RTX. In the DESIRES trial [19], which demonstrated the efficacy of RTX for skin sclerosis in SSc, FVC improved after 6 months of RTX treatment. This suggests that RTX may also be effective in treating SSc-ILD. In our study of patients with SSc-ILD, FVC increased in all patients in the NTB + IS (RTX) group, while in the NTB group, FVC increased in 4 out of 8 patients (50%) (Additional file 1). Based on these results, the combination of NTB and RTX could be a viable treatment option for SSc-associated PF-ILD, however the improvement in FVC may simply be because of RTX. The usefulness of the combination of NTB and RTX will not be seen until at least a comparison is made between the RTX monotherapy and NTB + RTX combination therapy.

The concomitant IS used by PM/DM patients in the NTB + IS group were GC, MMF, RTX, and cyclophosphamide. These have been reported as treatment options for PM/DM-ILD [6–10, 20–22]. All PM/DM patients in the NTB + IS group showed an increase in FVC, while none of the PM/DM patients in the NTB group did. Even though the number of patients with PM/DM-ILD was limited (three patients each), the combination of NTB and IS might offer more benefits than NTB alone in treating PM/DM-associated PF-ILD (Additional file 1).

Two patients with RA-ILD in the NTB + IS group received abatacept as the concomitant immunosuppressant. Although there are many reports on the efficacy of abatacept for treating RA-ILDs [23–25], there are limited reports on the combination of NTB and abatacept. In this study, FVC increased in both patients in the NTB + IS (abatacept) group. However, the efficacy of combining NTB and abatacept for treating RA-associated PF-ILD needs to be validated in a larger patient cohort.

The cause of the improvement in FVC in the NTB + IS group might be due to the additive effects of anti-inflammatory and anti-fibrotic agents, since both proinflammatory and profibrotic mediators are thought to be closely involved in the pathogenesis of CTD associated PF-ILD [6, 26]. However, the choice of IS to be started depend on the type of CTD and should be validated in a larger patient cohort. Additionally, the difference in efficacy between NTB + IS combination therapy and IS monotherapy for CTD-associated PF-ILD is needed to be clarified in the future.

The AEs associated with NTB in this study were similar to those reported in previous clinical trials [1]. There was no difference in the incidence of AEs between the NTB group and the NTB + IS group. In the NTB + IS group, one patient had gastrointestinal perforation, a serious AE. This patient, who had DM-ILD, developed a sigmoid diverticular perforation and had acute abdominal pain 4 months after starting NTB + IS (RTX) therapy. The patient had also been receiving GC for a long time before starting NTB treatment. The inhibition of VEGF by NTB has been reported to be associated with an increased risk of gastrointestinal perforation [27]. In the INPULSIS trial, which demonstrated the efficacy of NTB in patients with IPF, gastrointestinal perforation was reported in 0.3% of patients receiving NTB (vs. 0% in placebo group) [28]. Gastrointestinal perforation during NTB treatment may be a concern, especially in patients with CTD-associated PF-ILD who are on long-term GC therapy and taking nonsteroidal anti-inflammatory drugs, as these are known risk factors for gastrointestinal perforation [29].

In this study, serum KL-6 was assessed as a biomarker for ILD. Serum KL-6 levels are elevated in patients with CTD-ILD as well as IPF, and they correlate with the severity of CTD-ILD, as determined by HRCT and PFT, and serve as a biomarker for predicting progression [16, 30]. However, to the best of our knowledge, there is only one study that has examined changes in KL-6 levels during NTB treatment. This study reported that persistently elevated KL-6 levels during NTB treatment were associated with a reduction in FVC in patients with IPF [31]. In our study, serum KL-6 levels significantly decreased after NTB treatment, and a significant negative correlation was observed between changes in serum KL-6 (U/mL) and changes in FVC (%). This is the first report indicating that serum KL-6 levels could serve as a biomarker for NTB treatment response in patients with CTD-associated PF-ILD. Additionally, elevated serum KL-6 levels during NTB treatment could indicate a worsening of CTD-associated PF-ILD.

This study has several limitations. First, this study lacked a control group that did not receive NTB. Therefore, it was impossible to compare the outcomes of patients treated with NTB to those without NTB in evaluating the efficacy of NTB for CTD-associated PF-ILD. Since NTB has been approved as a standard of care for PF-ILD, it was ethically difficult to establish a control group. Nevertheless, our findings demonstrate the efficacy of NTB in slowing the progression of CTD-associated PF-ILD, as shown by a significant improvement in the monthly change in FVC (%/month) following NTB treatment. Second, the small number of patients in this study made it difficult to carry out a stratified analysis by each CTD. Third, selection bias is present in this study because of the retrospective monocenter design. Fourth, selection bias is also present in patient assignment because the decision to prescribe NTB alone or both NTB and IS was made by the attending physician following the diagnosis of CTD-associated PF-ILD. Therefore, there was a variation of CTDs in each group. However, there were no significant differences between the two groups in the proportion of UIP-like pattern on HRCT, the duration of ILD, and the proportion of patients who met each criterion for disease progression, thus the characteristics of PF-ILD were similar in both groups. Finally, 10 out of the 15 patients in the NTB group had received IS before being diagnosed with PF-ILD and continued on the same IS treatment after their diagnosis of PF-ILD (Additional file 1). Therefore, these patients in the NTB group may also have benefited from the NTB + IS combination therapy.

## Conclusions

This study demonstrated that NTB is an effective medication for slowing the progression of CTD-associated PF-ILD in real-world settings. The results also suggested that combination therapy with NTB and IS for CTD-associated PF-ILD may improve FVC.

### Electronic supplementary material

Below is the link to the electronic supplementary material.

Supplementary Material 1

## Data Availability

The data that support the findings in this study will be shared on reasonable request to the corresponding author.

## Abbreviations

AE: Adverse events
CTD: Connective tissue disease
EMA: European Medicines Agency
EULAR: European League Against Rheumatism
HRCT: High-resolution chest computed tomography
ILD: Interstitial lung disease
IPF: Idiopathic pulmonary fibrosis
PFT: Pulmonary function tests
RA: Rheumatoid arthritis
SD: Standard deviation
