# Supplementary material for: Nintedanib combined with immunosuppressive agents improves forced vital capacity in connective tissue disease-associated PF-ILD: a single-center study
Source: BMC Rheumatol. 2024 Jun 18;8:27. doi: 10.1186/s41927-024-00400-y (PMC11184786; doi:10.1186/s41927-024-00400-y)
Supplement: Supplementary file 1 — Supplementary Material 1 [file 41927_2024_400_MOESM1_ESM.docx]

Additional file 1. Details of each patient’s primary CTD type, pulmonary function test data, and immunosuppressive agents.

|  | Primary CTD | PFT at the start of NTB treatment | | Additional IS administered prior to the diagnosis of CTD-associated PF-ILD | Additional IS administered following the diagnosis of CTD-associated PF-ILD | PFT after NTB treatment | | |
| --- | --- | --- | --- | --- | --- | --- | --- | --- |
|  |  | FVC (%) | Monthly change in FVC (%/month) before NTB treatment |  |  | Follow-up period since NTB was started (months) | FVC (%) | Monthly change in FVC (%/month) after NTB treatment |
| NTB group | | | | | | | | |
| 1 | SSc | 46.2 | −0.05 |  |  | 12 | 54.3 | 0.67 |
| 2 | SSc | 67.9 | −0.69 | AZA, Tac |  | 9 | 66.9 | −0.11 |
| 3 | SSc | 59.2 | −0.4 | GC, CyA |  | 6 | 62.6 | 0.57 |
| 4 | SSc | 60.3 | −0.62 | GC, AZA | - | 6 | 57.5 | −0.47 |
| 5 | SSc | 55.1 | −0.67 | - | - | 7 | 57 | 0.27 |
| 6 | SSc | 75.4 | −0.1 | - | - | 6 | 73.4 | −0.33 |
| 7 | SSc | 55.3 | N/A | GC | - | 10 | 56.1 | 0.08 |
| 8 | SSc | 74 | −0.02 | - | - | 6 | 72.3 | −0.28 |
| 9 | DM | 61.9 | N/A | GC, AZA | - | 6 | 47.3 | −2.43 |
| 10 | DM | 83.9 | −0.34 | GC, Tac | - | 8 | 77.3 | −0.83 |
| 11 | DM | 107.8 | N/A | GC | - | 6 | 105.3 | −0.42 |
| 12 | RA | 48.6 | −0.58 | GC, Tac | - | 12 | 45.2 | −0.28 |
| 13 | RA | 48.4 | N/A | GC, Tac | - | 6 | 58 | 1.6 |
| 14 | EGPA | 34.4 | −1.21 | GC, Mepo | - | 12 | 31.9 | −0.21 |
| 15 | SS | 85.6 | −0.38 | - | - | 9 | 82.8 | −0.31 |
| NTB + IS group | | | | | | | | |
| 1 | SSc | 80.5 | −1.73 | GC, Tac | RTX | 6 | 83.6 | 0.52 |
| 2 | SSc | 52.8 | N/A | GC, AZA | RTX | 11 | 72.5 | 1.79 |
| 3 | SSc | 57.6 | N/A | GC, AZA | RTX | 12 | 84.7 | 2.26 |
| 4 | DM | 42.4 | −0.68 | GC, Tac, MMF | RTX | 8 | 50.8 | 1.05 |
| 5 | DM | 64.1 | −0.78 | GC, CyA | GC, CyA→MMF | 10 | 69.9 | 0.58 |
| 6 | PM | 60.3 | N/A | - | GC, CY, Tac | 6 | 99.7 | 6.57 |
| 7 | RA | 84.6 | −1.09 | Tac | Tac→ABT | 7 | 91 | 0.91 |
| 8 | RA | 57.6 | −2.1 | GC, Tac | ABT | 9 | 62 | 0.49 |
| 9 | MPA | 70.7 | −0.95 | GC | GC, RTX | 11 | 68.2 | −0.23 |
| 10 | MPA | 46.4 | −0.17 | GC, MMF | GC | 8 | 55.7 | 1.16 |
| 11 | MPA | 44.5 | N/A | GC | RTX | 9 | 56.7 | 1.36 |

CTD: connective tissue disease; PFT: pulmonary function test; IS: immunosuppressive agents; FVC: forced vital capacity; NTB: nintedanib; SSc: systemic sclerosis; DM: dermatomyositis; RA: rheumatoid arthritis; EGPA: eosinophilic granulomatosis with polyangiitis; SS: Sjögren’s syndrome; PM: polymyositis; MPA: microscopic polyangiitis; AZA: azathioprine; Tac: tacrolimus; GC: glucocorticoids; CyA: cyclosporine A; Mepo: mepolizumab; MMF: mycophenolate mofetil; RTX: rituximab; CY: cyclophosphamide.
